# Influence of Gravitational Orientation on the Formation of Electrodeposited Copper Wicks

**DOI:** 10.3390/ma19214539

**Published:** 2026-10-28

**Authors:** Oliver Skura, Mateusz Boćko, Pawel Szymanski

**Affiliations:** Faculty of Mechanical Engineering and Ship Technology, Institute of Energy, Gdańsk University of Technology, Narutowicza St. 11/12, 80-233 Gdańsk, Poland

**Keywords:** porous media, capillary transport, electrodeposited copper wick, gravitational effects, liquid transport

## Abstract

**Highlights:**

- Twenty-four copper wicks were fabricated by the electrochemical deposition method.
- Wicks were formed in Top, Bottom, and Vertical orientations relative to gravity.
- Top-oriented wicks achieved 37–45% greater capillary lift heights.
- Bottom and Vertical configurations showed similar mean capillary transport performance.
- Gravitational orientation should be considered as a parameter during electrochemical wick fabrication.

**Abstract:**

Porous wicks ensure capillary liquid transport in heat pipes and vapour chambers. Manufacturing them through electrochemical deposition is a promising method that yields competitive results. The gravitational orientation during electrochemical deposition, however, has rarely been taken into consideration. In this study, the effect of deposition orientation relative to gravity on the capillary performance of electrodeposited copper wicks was investigated. In total, 24 samples were prepared in three configurations defined by the angle between the cathode surface normal and gravitational acceleration: Top (22.8°, downward-facing), Vertical (90°, sideways-facing), and Bottom (157.2°, upward-facing). All other electrodeposition conditions were kept identical. Capillary performance was evaluated for 5 samples per configuration using a capillary lift rate test. The Top configuration achieved up to 44.8% greater mean capillary lift height than the Bottom configuration after 5 s. The Bottom and Vertical configurations exhibited similar capillary rise behaviour, whereas Top samples reached 15 mm in 19.4 s, compared with 42.9 s and 41.4 s for the Bottom and Vertical configurations, respectively. These results suggest that gravitational orientation affects the capillary performance of electrodeposited copper wicks and should be considered during their fabrication.

## 1. Introduction

The continuing miniaturisation and increasing power density of electronic components create demanding thermal requirements. Among the technologies used to satisfy these requirements are heat pipes (HPs) and vapour chambers (VCs), which are passive two-phase heat transport equipment used to transport thermal energy between designated regions of a system. During operation, heat is supplied to the evaporator section of a HP, where it is absorbed by the working fluid and converted into vapour. The resulting pressure difference between the evaporator and condenser sections drives the vapour towards the condenser section, where it releases its energy in the form of heat and returns to the liquid state. The working fluid is subsequently transported back to the evaporator section through the porous wick by capillary action, completing the cycle [1]. Consequently, improving the capillary transport capability of wick structures in HPs continues to be an important area of research. Traditional wick architectures include grooved surfaces, wire meshes, and sintered-powder structures. Their capillary performance is constrained by the opposing effects of pore size on permeability and capillary pressure; larger pores facilitate a low-resistance liquid flow but generate lower capillary pressure, whereas smaller pores provide stronger capillary pumping at the expense of permeability [2].

Among the fabrication methods investigated for such structures, electrochemical deposition offers a means of forming thin, porous copper wicks with modifiable morphology directly on metal substrates [3,4]. Early work showed that the resulting wick performance strongly depends on the deposition process. Lu et al. [5], for example, demonstrated that deposition current density as well as time affected both permeability and capillary performance of a porous layer deposited on rectangular grooves, with the modified capillary-performance parameter reaching its maximum at 20 min and 50 mA cm^−2^. Subsequent studies increasingly focused on hierarchical and dendritic structures. Luo et al. [6] developed a one-step electrodeposited biomimetic copper-forest wick and successfully implemented it in a 0.6 mm-thick HP, while Yu et al. [4] showed that the morphology and capillary properties of dendritic copper wicks could be controlled through changes in current density and ${\mathrm{CuSO}}_{4}$ concentration. Other studies demonstrated that substrate topography [2] and the combination of dendritic structures with conventional mesh wicks [7] also impact liquid transport, while Chiang et al. [8] demonstrated the applicability of hierarchical dendritic wicks in high-power VCs.

The formation of electrodeposited copper structures begins with the nucleation and subsequent growth of copper deposits on the substrate surface. Grujicic and Pesic [9] demonstrated that the size and population density of copper nuclei on glassy carbon substrates depend on electrolyte composition, pH, and deposition potential. For copper substrates, Liu et al. [2] showed that the initial surface topography influences the nucleation and growth of dendritic copper deposits, affecting their subsequent morphology and capillary performance. At sufficiently high electrode overpotentials, hydrogen evolution can accompany copper electrodeposition on the surface of the cathode. The formed bubbles can temporarily mask the substrate surface while copper is deposited on the exposed conductive regions around them. Bubble growth, coalescence, motion and detachment can consequently influence the resulting porous morphology [4,10,11]. Li et al. [10] demonstrated that increasing the deposition current density from 0.1 A cm^−2^ to 1.2 A cm^−2^ reduced the average pore diameter from 150 μm to 50 μm, illustrating the influence of deposition conditions on bubble-templated pore morphology. He et al. [12] further demonstrated that variations in hydrogen evolution during copper electrodeposition influence both the resulting wick morphology and its capillary performance.

Beyond electrochemical parameters, physical conditions that influence hydrogen bubble behaviour may also affect the resulting deposit morphology. Attia et al. [13] recently showed that electrode orientation during dynamic hydrogen bubble templating altered the pore structure and dendritic morphology of electrodeposited copper foams. However, their study focused on morphological control and electrochemical applications of the foams rather than their capillary transport behaviour. This leaves open the question of whether orientation-induced morphological differences during electrochemical deposition subsequently affect the capillary performance of copper wick structures.

Accordingly, this work focuses on the effect of gravitational orientation during electrochemical deposition on the capillary performance of copper wick structures. Samples were fabricated under otherwise identical conditions in three configurations relative to gravity: upward-facing, downward-facing, and vertical, and their subsequent liquid transport behaviour was evaluated using capillary lift height experiments. The objective is to identify whether deposition orientation constitutes a relevant manufacturing parameter for wick structures intended for differently oriented surfaces in three-dimensional VC geometries, while giving insight into the gravity-dependent nature of the deposition process.

## 2. Materials and Methods

### 2.1. Sample Preparation and Electrochemical Deposition

**Figure 1:**
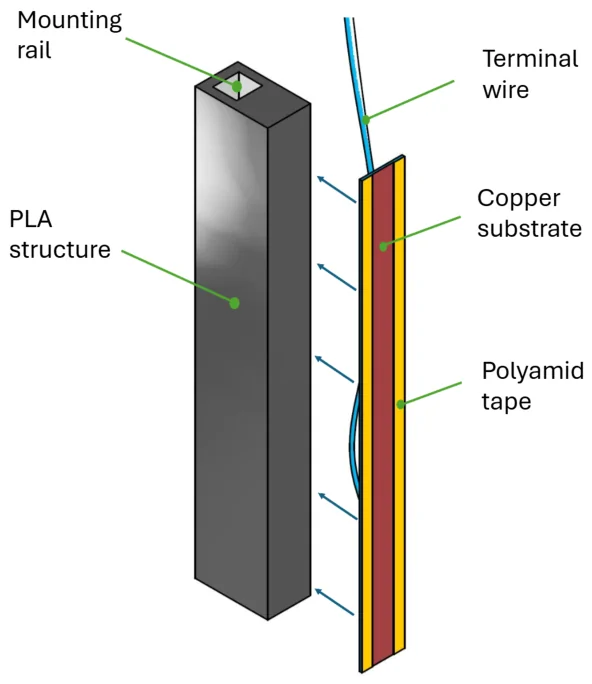
Assembly of a sample.

In this study, 24 samples were prepared from copper plates with dimensions of 100 mm · 10 mm · 0.5 mm. Each plate was connected to a copper terminal wire by soldering using tin as the soldering material. The connected face, together with most of the attached wire, was coated with an electrically insulating acrylic-resin layer. The sample was then assembled onto a PLA (polylactic acid) structure using epoxy resin, as shown in Figure 1. The exposed copper surface was subsequently mechanically prepared using a 220-grit abrasive to ensure surface flatness, remove oxidation and foreign material, and provide a microscopically rough surface. These resulting microscopic grooves were intended to serve as a basis for the nucleation of expected dendritic structures. The samples were split equally among the 3 intended configurations: Top, Bottom and Vertical. Deposition of the porous structure was performed on every one of the 24 samples. 5 samples from each configuration were selected to be tested and included in the results. The remaining 3 samples out of each configuration were marked as spares. The spares consisted of samples which included defects from the deposition procedure or handling between the different steps, or capillary rise tests performed under altered conditions.

Subsequently, the long edges of the exposed copper plates were covered with polyamide tape until a 5 mm-wide strip of copper remained exposed along the full length. The sample was then mounted opposite to a copper anode, prepared in the same manner, such that the two exposed copper surfaces were parallel and separated by a distance of (25 ± 1) mm. An adapter was fabricated to enable the electrodes to be positioned at different orientations. The assembly was then submerged in a copper sulfate electrolyte solution in the desired configuration.

**Figure 2:**
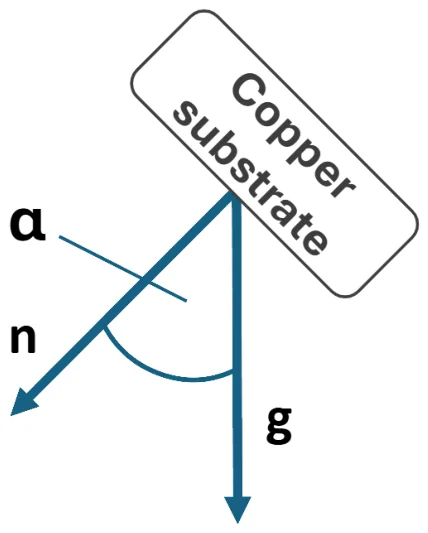
Definition of the angle used in any configuration, with **a** being the angle, **g** being the vector of gravitational acceleration and **n** being the normal vector of the copper substrate surface.

**Figure 3:**
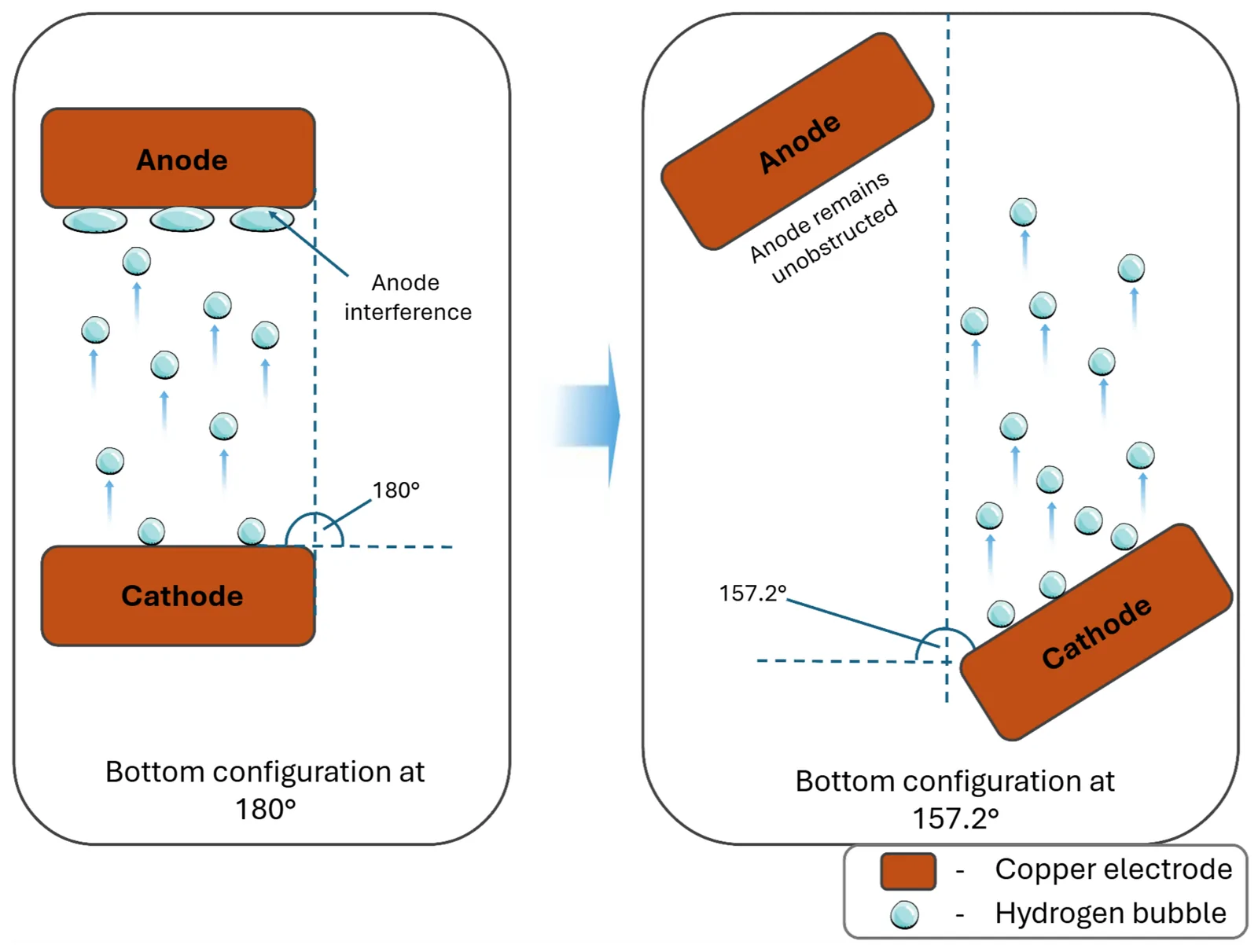
Behaviour of formed hydrogen bubbles during electrochemical deposition at angles of 180° and 157.2° in the Bottom configuration.

Table 1 summarises the investigated configurations and their respective orientations relative to gravity. The focus of this study lies on the analysis of any differences between surfaces being the “floor”, “wall” and “ceiling” of a potential heat transport component with regard to the manufacturing parameters of an electrodeposited copper wick. Figure 2 illustrates the definition of the angle, describing the orientation of the copper substrate in a gravitational field. The normal positive vector of the substrate surface is being compared to the vector of gravitational acceleration. The angles were derived from the geometry of the samples and the setup with an additional safety margin. These angles were selected to reduce the risk of hydrogen bubbles interfering with the parallel anode during the deposition process. For example, placing the cathode directly underneath the anode caused hydrogen bubbles to rise and stick to the surface of the anode, thus preventing a substantial amount of its surface from being exposed to the solution. Figure 3 shows the setup for electrochemical deposition for the Bottom configuration at a nominal orientation of 180° and in a suitable rotated position of 157.2°. For the Top position, in which the cathode was placed directly above the anode, a different potential source of interference occurred in which parts of the deposited porous material could break off during the deposition process, sink under gravity, and collide with the anode, thus potentially disturbing the process.

Electrochemical deposition was performed in an electrolyte containing 0.8 mol L^−1^ CuSO_4_ and 1.5 mol L^−1^ H_2_SO_4_, at a current density of 0.5 A cm^−2^, a temperature of 25 °C and a deposition time of 1 min. A dendritic copper structure was thereby deposited on the exposed copper surface. Following this deposition and removal of the protective polyamide tape, the sample was rinsed with distilled water and dried in a vacuum chamber. It remained under a partial vacuum of 15 mbar until required. Each batch of 8 samples per configuration was deposited sequentially, with the vertical configuration being prepared first, followed by the Top configuration and concluded with the samples using the Bottom configuration.

### 2.2. Capillary Lift Rate Experimental Setup

**Figure 4:**
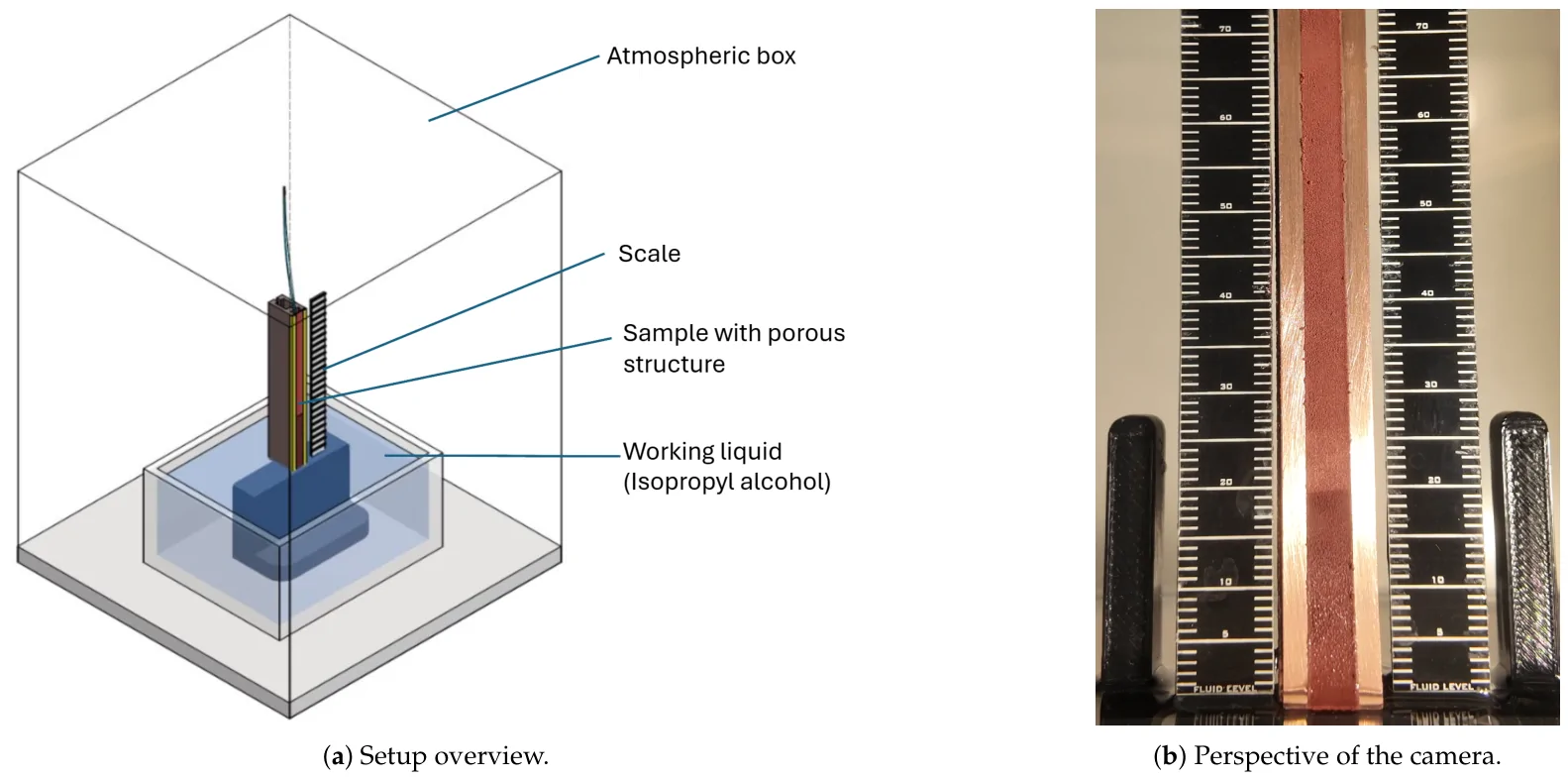
Setup of the capillary lift height rate test.

To obtain quantitative results from the samples, a capillary lift rate test was performed. As seen in Figure 4, the test involves partially immersing a sample in a working liquid and allowing the liquid to rise along the wick against gravity by capillary action. The momentary height of the averaged liquid front was captured at fixed temporal intervals. For each test, a dried sample is lowered onto a fixture which is submerged in a liquid (isopropyl alcohol). The moment the sample touches the liquid film and the alcohol starts climbing up the wick, its colour changes drastically from a light copper orange to a dark one, which can be observed and documented using an appropriate optical camera recording a high-resolution video at a framerate of 59.94 frames per second. The perspective of the optical system is displayed in Figure 4. To facilitate accurate measurements of the current height of the liquid film, two scales with a resolution of 1 mm were positioned next to the sample. This process was repeated twice for each analysed sample. For the evaluation of the results, only the first lift rate test for every sample was taken into account. The second test was performed to verify the repeatability of the capillary rise behaviour. During the capillary rise test, the repeated tests lagged behind the primary test by 0–2 mm.

An image-processing algorithm was used to detect the height of the liquid front and associate it with a timestamp extracted from the corresponding video frame, allowing the different samples to be compared regarding their performance in pumping the working liquid against gravity. To synchronise the corresponding camera footage, the videos were temporally aligned using the first frame in which a liquid front extending above the fluid level was manually identified. To minimise any evaporation of the liquid on the wick while it is rising, the entire experimental setup was contained inside a chamber filled with an atmosphere saturated with the vapour of the working liquid in use. The camera observed the samples through a transparent viewport. To minimise parallax errors from the perspective of the camera, the substrate, including the porous copper structure, was positioned on the same plane as the two scales in use. Given the resolution of the scales of 1 mm, the uncertainty of measurement was estimated at ±0.50 mm.

As a supplementary test, one sample from each configuration was left in the capillary lift rate test for 1 h. All samples tested pumped the working liquid to their full height of 100 mm during the elapsed time. Consequently, this test did not provide sufficient information to determine the maximum capillary lift height.

Further morphological data have been acquired by preparing samples for cross-sectional optical microscopy and by recording their deposition process with a suitable optical capturing system.

### 2.3. Image Processing and Data Analysis

**Figure 5:**
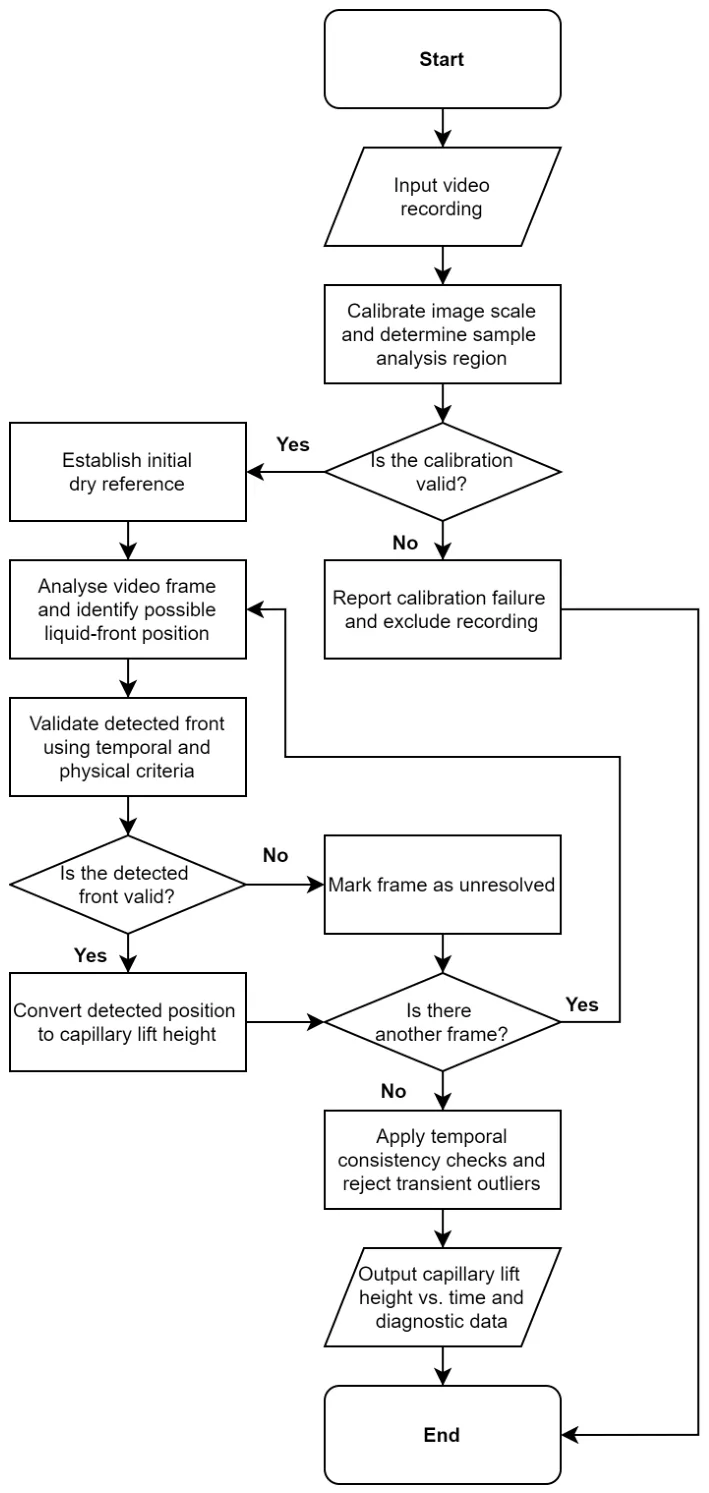
Simplified workflow of the image-processing and data-analysis procedure used to determine capillary lift height from the recorded experiments.

The measurements were processed using a custom image-analysis algorithm implemented in Python (version 3.14.5) [14]. The input to the algorithm consisted of video recordings of the experiments, each lasting 45 s, in which the tested copper wick sample was positioned between two graduated reference rulers. Each recording was visually inspected to verify that the initial liquid contact corresponded to the first analysed frame, defined as t=0. Subsequent timestamps were calculated from the frame indices and the recorded frame rate. The objective of the algorithm was to automatically determine the position of the advancing liquid front in successive video frames and convert this position into a physical capillary lift height as a function of time. The algorithm used OpenCV (version 4.14.0) [15] and NumPy (version 2.5.2) [16] for image processing and numerical operations, together with Matplotlib (version 3.10.9) [17] for plot generation. For each analysed recording, the principal output was a time-resolved series of capillary lift height in mm. Additional outputs included diagnostic images showing the detected geometry and liquid-front tracking, plots of capillary lift height versus time, a marked video for visual verification of the detection process, and CSV files containing the measured height–time data. Replicate recordings belonging to the same configuration were subsequently combined to obtain mean curves and associated statistics (e.g., standard deviation or the number of contributing measurements). The image-processing workflow applied to each individual recording is summarised in Figure 5.

Before any front detection, the geometry of each recording was calibrated automatically. An early video frame was selected in which both reference rulers and their major scale markings could be detected reliably. Corresponding ruler marks on the left and right sides of the sample were paired and used to establish the physical vertical scale, with intermediate 1 mm reference positions obtained by spatial interpolation. The outlines of the copper sample were then detected, allowing its centreline to be defined. The calibrated ruler positions were referenced to this centreline to establish the relationship between vertical image position and physical lift height in mm. The initial liquid level was defined as the 0 mm reference position. For each video frame, the median lightness across the analysed sample width was calculated from the lightness channel of the CIELAB colour representation [18], producing a one-dimensional brightness profile along the wick height. For inclined or uneven wetting fronts, this approach determines a representative height based on the lightness transition rather than the highest wetted point or the arithmetic mean of local front heights. The profile was spatially smoothed, and locations exhibiting pronounced changes in lightness were identified as potential liquid-front positions. Several candidate positions were retained in each frame because surface texture, reflections, and other optical features could also produce strong image edges.

The candidate front positions were subsequently evaluated using temporal changes in the appearance of the sample. The first video frame was used as the reference state, while the first 5 frames were used to estimate the normal level of image noise. The estimated noise level was subsequently used to adapt the threshold required for accepting temporal changes as evidence of wetting. For each candidate position, the change in lightness relative to the reference frame was compared in regions immediately above and below the candidate. A candidate was accepted either when the temporal change across the front provided sufficiently strong evidence of wetting or when a relative change was additionally supported by continuity of the changed region towards the liquid reservoir. This additional connectivity condition was used to reject stationary surface features, reflections, and other local image changes that did not correspond to the advancing wetting front. Specifically, a dedicated procedure was used to reduce false detections caused by the liquid meniscus and strong reflections close to the initial liquid level.

Finally, newly detected front positions were subjected to a multi-frame confirmation procedure. A new capillary lift height level was accepted when at least three of 5 consecutive frames contained sufficiently confident detections within a tolerance of ±0.50 mm. Isolated excursions were treated as transient detection artefacts and rejected. In this context, rejected or unresolved measurements were not replaced by interpolated or reconstructed values; only positions derived directly from actual video frames were retained in the final measurement series. For replicate recordings corresponding to the same experimental configuration, the final measured height series were combined frame by frame. At each time point, only available frame-derived measurements were included in the calculation of the mean capillary lift height and its standard deviation. The resulting mean curves were used for comparison between the investigated sample orientations.

## 3. Results

### 3.1. Morphological Observations

**Figure 6:**
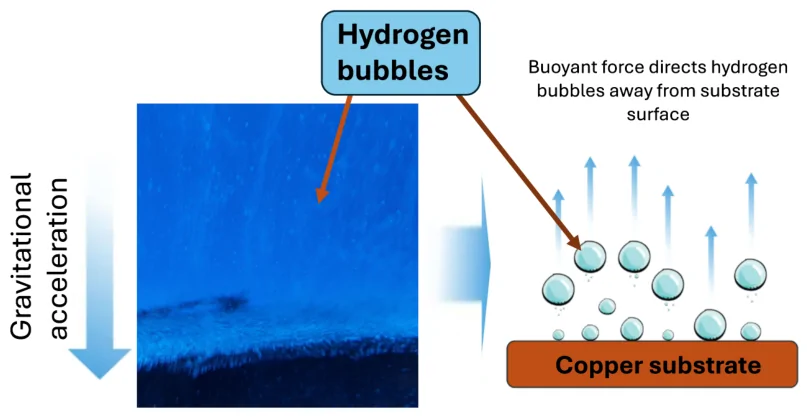
Behaviour of hydrogen bubbles during electrochemical deposition on the Bottom configuration.

**Figure 7:**
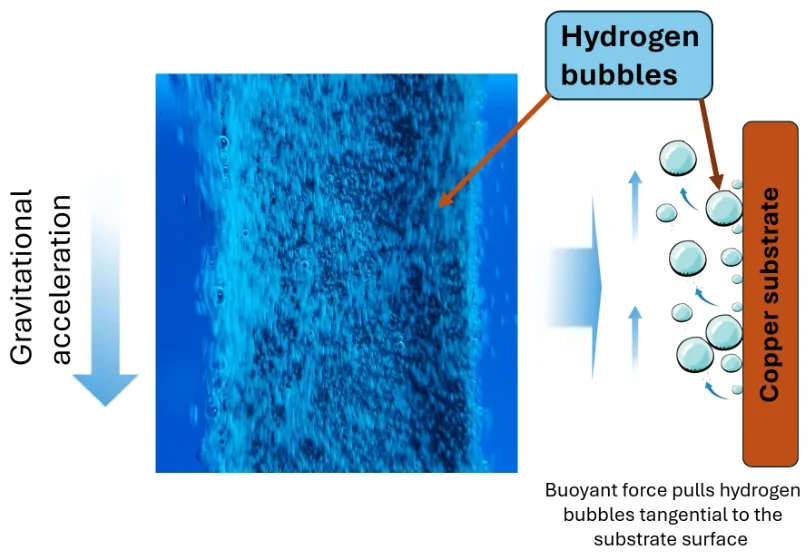
Behaviour of hydrogen bubbles during electrochemical deposition in the Vertical configuration.

**Figure 8:**
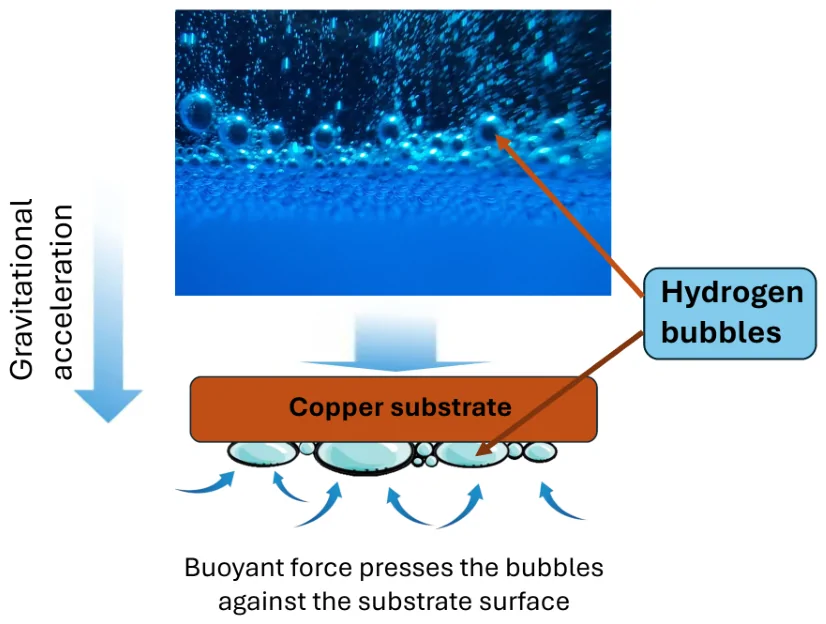
Behaviour of hydrogen bubbles during electrochemical deposition on the Top configuration.

Figure 6, Figure 7 and Figure 8 illustrate the behaviour of the hydrogen bubbles formed during the process based on the qualitative review of the video footage of the deposition process. In the Bottom configuration, these bubbles tend to grow to a certain size before detaching from the surface, leaving the dynamic template. The Vertical configuration also promotes bubble detachment from the surface, as in the Bottom configuration. However, the bubbles formed and released from lower regions appear to collide with ones formed above them, assisting in their removal. This creates a highly dynamic environment with pronounced levels of liquid agitation. The Top configuration differs substantially, as the formed bubbles were pushed against the substrate surface. Due to the continuing formation of hydrogen, the bubbles grew in size, enabling their dimensions to grow noticeably larger than those observed in the Bottom and Vertical configurations.

**Figure 9:**
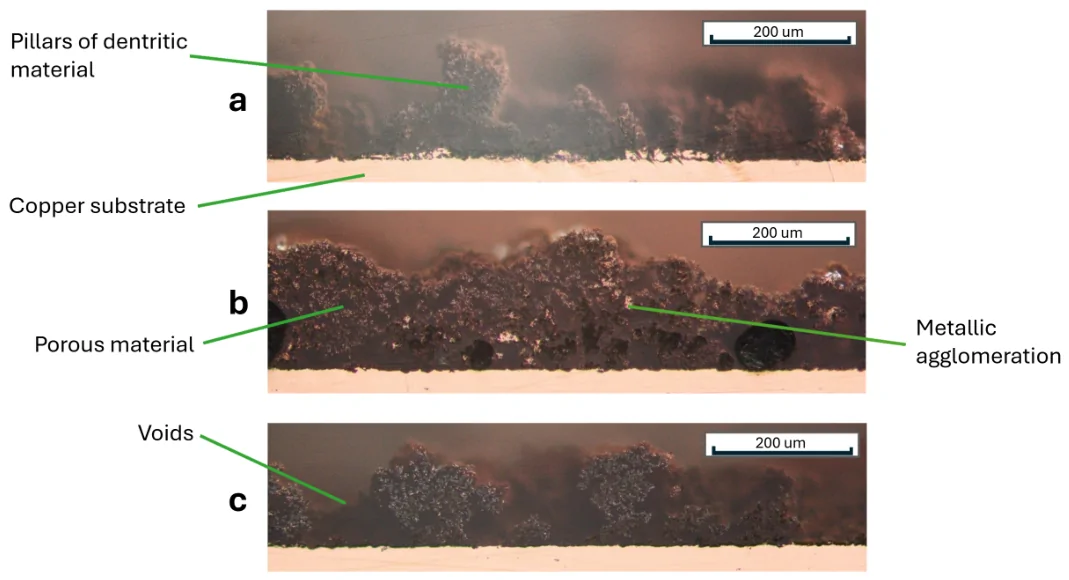
Cross-sectional microscopy of the samples: (**a**) Top configuration, (**b**) Vertical configuration, (**c**) Bottom configuration.

Figure 9 represents a cross-sectional view of the porous structures deposited on the substrate surfaces. The thickness of the structures fluctuates between 100 μm and 200 μm. Upon closer inspection, the structures can be identified as dendritic copper “bushes” being created through electrochemical deposition as described by Yu et al. [4]. On the sample produced using the Top configuration, the structures appear to feature predominantly singular dendritic pillars with shorter pillars distributed in between. The empty regions between these adjacent pillars can occasionally almost extend to the original copper surface. Some deposited agglomerations of dense copper metal can be spotted close to the original copper surface. The sample produced using the Vertical configuration displays a largely uniform layer of dendritic bushes. It forms a dense “forest” and rarely decreases its thickness below 100 μm. Furthermore, several agglomerations can be seen inside the wick. These agglomerations appear to be localised growth spots of copper.

**Figure 10:**
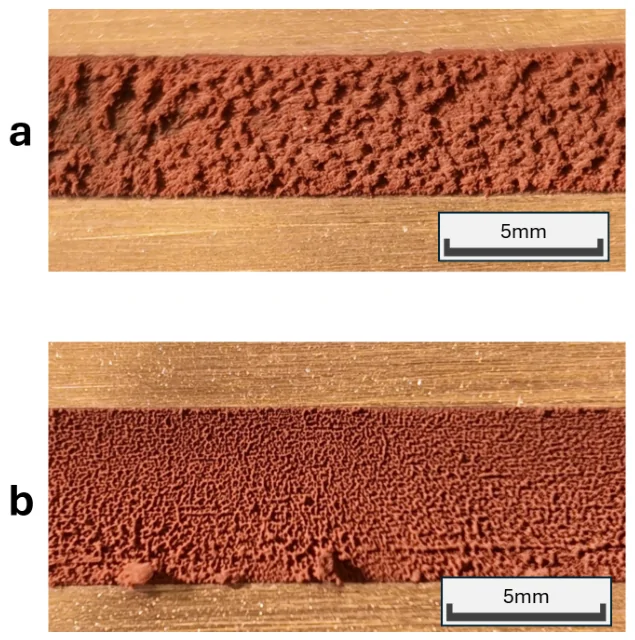
Comparison of two samples from the Vertical (**a**) and Top configurations (**b**).

The sample of the Bottom configuration features, just like the Top configuration, pillar-like structures being interrupted by noticeable voids. The gaps are noticeably smaller than on the Top configuration. Comparing the two samples, as demonstrated in Figure 10, reveals that the wick from the Vertical configuration behaves like a dense structure being interrupted by an occasional small void, while the one from the Top configuration consists of thin pillars surrounded by large voids.

### 3.2. Capillary Lift Height Behaviour

**Figure 11:**
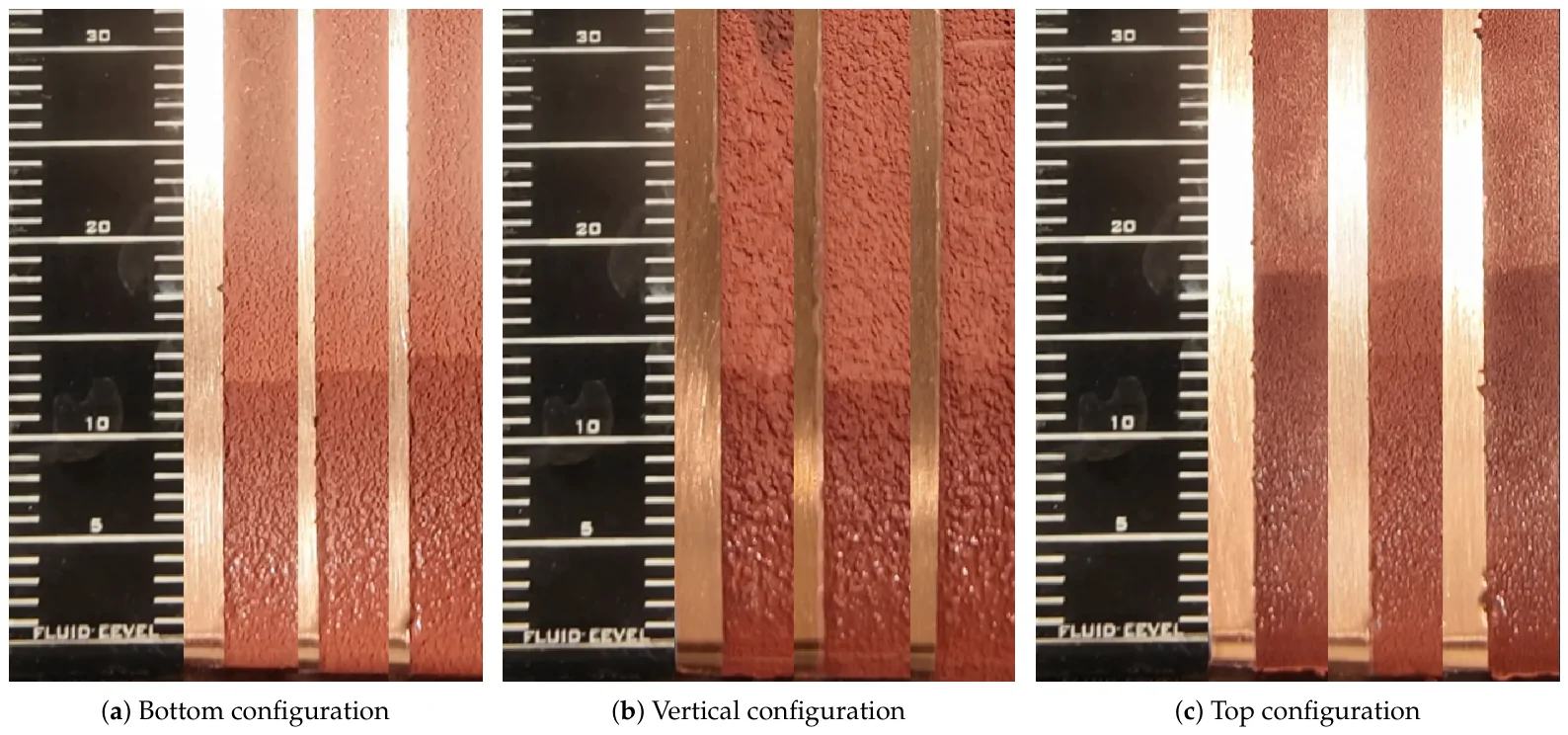
Comparison of the lift height of all three configurations at 30 s.

Figure 11 displays several samples from different configurations as viewed by the high-resolution camera. After 30 s, the Top configuration reached an average capillary height of 18 mm, the Vertical configuration 13.2 mm, and the Bottom configuration 13.0 mm. Owing to the limited resolution of the scales, the measurements were assigned an uncertainty of ±0.50 mm. The capillary lift rate test was repeated after complete dry-out of the sample to assess the repeatability of the results.

Table 2 compares the mean capillary lift height at selected time stamps. All values featured represent the averages of samples from the same configuration. The table analyses the effective speed of the liquid inside the wick in relation to the distance covered after a specific time. Notably, all five specimens contributed to each reported value, except for the Top configuration at 45 s (n=4).

### 3.3. Effect of Electrochemical Deposition Orientation on Capillary Performance

**Figure 12:**
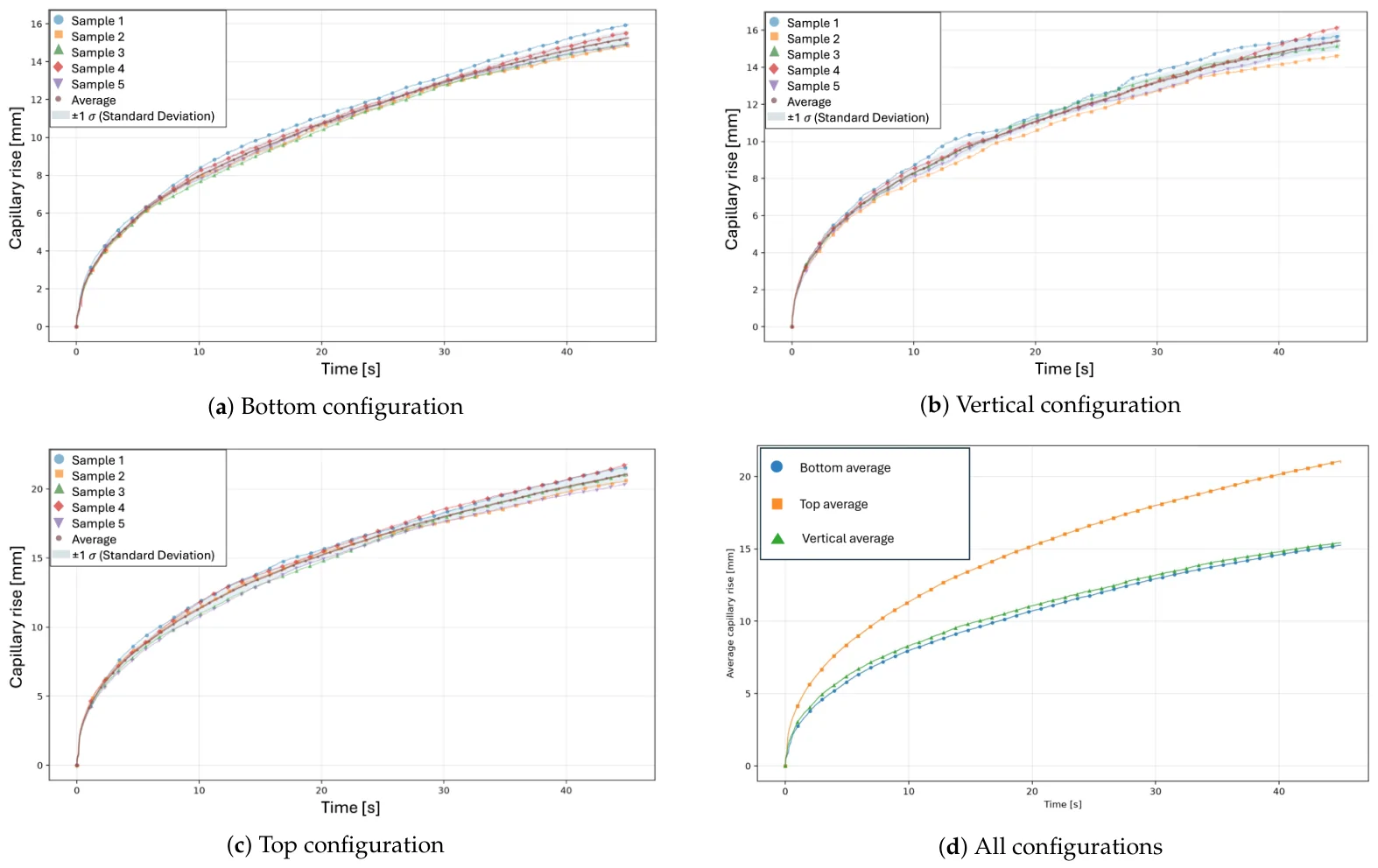
Capillary lift height as a function of time for the (**a**) Bottom, (**b**) Vertical, and (**c**) Top configurations, showing individual samples, mean curves, and standard deviations; (**d**) comparison of the mean curves for all three configurations.

Figure 12 displays the results of the image-processing algorithm measuring the lift height over time. Each configuration was analysed using 5 samples, with any additional samples per configuration reserved as spares. Across all three configurations, 68 out of 40,470 potential specimen-frame measurements (0.17%) remained unresolved and were excluded from the statistical analysis. The maximum standard deviations were 0.44 mm, 0.52 mm, and 0.55 mm for the Bottom, Vertical, and Top configurations, respectively. Comparing all averages together indicates quantitatively that the Top configuration differs clearly from the Bottom or Vertical configuration. By assessing the difference in lift height after a given elapsed time, the Top configuration exhibits higher capillary performance than the Bottom configuration by up to 44.8% as shown in Table 2.

To compare the configurations at a fixed capillary lift height of 15 mm, the effective average rise speed was defined as the target height divided by the mean time required to reach it. The percentage increase in effective average rise speed was calculated as $\Delta v_{\mathrm{eff}}\left[\%\right]=100\left({\bar{t}}_{\mathrm{ref}}/{\bar{t}}_{\mathrm{Top}}-1\right)$, where ${\bar{t}}_{\mathrm{ref}}$ and ${\bar{t}}_{\mathrm{Top}}$ denote the mean times required to reach the target height for the reference and Top configurations, respectively. Accordingly, the Top configuration achieved 113.4% and 121.1% higher effective average rise speeds than the Vertical and Bottom configurations, respectively. To reach 15 mm in height, the Vertical and Bottom configurations require, on average, 41.4 s and 42.9 s, respectively. The samples using the Top configuration reached on average 15 mm after 19.4 s. In contrast, the Bottom and Vertical configurations exhibited similar mean capillary rise behaviour throughout the measurement period.

## 4. Discussion

### 4.1. Effect of Electrochemical Deposition Orientation on Wick Performance

The quantitative results reveal a large separation in capillary lift rate between the Top configuration and the remaining two configurations. This experiment shows that the orientation of the deposition surface influences the resulting capillary performance. Based on the mean times required to reach 15 mm in lift height, the Top configuration exhibited an effective average rise speed more than 100% higher than either the Bottom or Vertical configuration. This could yield unexpected results if a VC is to be coated with an electrodeposited wick on more than one face simultaneously. These results identify the gravitational orientation as a relevant fabrication parameter for electrodeposited wick structures. The maximum lift height could not be determined, as the samples used in the supplementary tests reached the full height of 100 mm during the capillary rise test and no observable state of equilibrium could be reached.

### 4.2. Relationship Between Wick Morphology and Capillary Performance

The cross-sectional microscopy shows that the porous structure in the Vertical configuration reached 200 μm in thickness, while the structures in Top and Bottom configurations only reached a maximum porous structure thickness of approximately 150 μm. Despite the large difference in structure height, the Bottom and Vertical configurations performed nearly identically during the capillary lift rate test. Without an observed and documented capillary equilibrium, it is unclear if, despite the similar capillary lift rate, the maximum capillary lift height could differ. Furthermore, the observed difference in capillary lift rate may be associated with orientation-induced differences in wick morphology, such as the relationship between permeability and capillary pressure which, together with the surface wettability, have not been independently determined in this study, but may affect the capillary performance. Any differences in qualitative observations provide a possible explanation, as the Top configuration appears to have larger voids between its dendritic pillars; the featured morphology may provide lower-resistance pathways for liquid transport, which could contribute to the observed capillary rise behaviour. In contrast, the finer structures observed in the other configurations may provide stronger capillary pumping pressure while exhibiting greater resistance to liquid flow. The different structures may be explained by the formed hydrogen bubbles, which form a dynamic template, as described in [13]. In the Top configuration, these bubbles were qualitatively observed to remain on their respective substrate surface comparably longer, potentially allowing them to grow larger and displace a greater surface area, thereby contributing to the formation of a less dense dendritic structure with larger voids. In the Vertical and Bottom configurations, bubbles were observed to detach from the electrode surface more readily and therefore to be generally limited to growing to a small size. The more frequent exchange of smaller bubbles may contribute to the formation of a more homogeneous, finer wick structure.

### 4.3. Future Work

Although differences in samples produced by the three investigated configurations could be shown, quantitative characterisation could be improved by the use of advanced microscopy and the analysis of average pore size or hydrophilicity. While the present study focused on the capillary lift rate, an analysis of the maximum capillary lift height could yield further valuable results. Similarly, the influence of different working fluids, particularly water, on the observed orientation-dependent capillary behaviour could be investigated. Moreover, these observations raise the question of whether reducing gravitational effects would decrease the orientation dependence observed during electrochemical deposition. Since the configuration in which, based on qualitative observations of the deposition process, the bubbles appear to stay the longest, thus potentially being able to grow largest, outperforms the remaining ones in the capillary lift height test, this leads to the proposal of further experiments to investigate whether forming wick structures under microgravity conditions improves their performance. Under microgravity conditions, reduced buoyancy-driven bubble detachment could keep bubbles attached to the electrode surface for longer and alter bubble growth together with their coalescence behaviour. Investigating these effects could therefore provide insight into whether reduced gravity modifies the morphology and capillary performance of electrodeposited copper wicks.

## 5. Conclusions

In this study, dendritic copper wick structures have been formed onto copper substrates using electrochemical deposition processes. To analyse the effect of the gravitational orientation on the resulting performance of the copper wicks, these samples have been formed in different configurations facing towards the gravitational acceleration (Top), away from it (Bottom) and perpendicular to it (Vertical). Using a capillary lift rate test, the Top configuration yielded an increase of up to 121.1% in the effective average rise speed to reach a vertical height of 15 mm from the liquid surface. The results demonstrate that the deposition orientation affects the capillary transport performance of electrodeposited dendritic copper wicks, indicating that gravitational orientation should be considered as a fabrication parameter.

## Figures and Tables

**Table 1:** Investigated configurations and their angles.

Configuration	Angle α [°]	Location of Substrate Surface
Top	22.8	located above the anode, facing downwards.
Vertical	90	placed vertically on the same level as the anode.
Bottom	157.2	located beneath the anode, facing upwards.

**Table 2:** Average capillary lift height for different sample configurations at selected times, including standard deviation.

Time, s	Top (SD), mm	Vertical (SD), mm	Bottom (SD), mm	Largest Difference, %
5	8.4 (0.34)	6.2 (0.17)	5.8 (0.11)	44.8
10	11.3 (0.46)	8.3 (0.3)	8.0 (0.26)	41.3
15	13.5 (0.38)	9.9 (0.38)	9.4 (0.28)	43.6
20	15.2 (0.34)	11.1 (0.28)	10.7 (0.23)	42.1
25	16.7 (0.27)	12.1 (0.27)	11.8 (0.19)	41.5
30	18.0 (0.38)	13.2 (0.4)	13.0 (0.17)	38.5
35	19.1 (0.45)	14.1 (0.39)	13.8 (0.29)	38.4
40	20.2 (0.45)	14.8 (0.43)	14.6 (0.36)	38.4
45	21.0 (0.53)	15.4 (0.51)	15.3 (0.43)	37.3

## Data Availability

The original contributions presented in this study are included in the article. Further inquiries can be directed to the corresponding author.
